# Grammatical metaphor studies: historical review and outlook

**DOI:** 10.3389/fpsyg.2026.1774523

**Published:** 2026-03-27

**Authors:** Yanchun Yu, Tianhua Wang

**Affiliations:** 1 School of Foreign Languages, Harbin University, Harbin, China; 2 School of Foreign Languages and Literature, Heilongjiang University, Harbin, China

**Keywords:** grammatical metaphor, historical review, ideational grammatical metaphor, interpersonal grammatical metaphor, outlook

## Abstract

Grammatical metaphor, a significant theoretical innovation within Systemic Functional Linguistics (SFL), extends meaning realization from the lexical to the grammatical stratum, providing a powerful framework for analyzing abstraction and technicality in academic and scientific discourse. Adopting a methodology of combining systematic retrieval with thematic analysis, this article reviews 293 primary studies on grammatical metaphor within SFL since Halliday initial proposal. We first trace Halliday’s three-stage theoretical development of grammatical metaphor. Subsequently, our thematic synthesis reveals predominant sub-themes within two major streams: theoretical development (encompassing semantic and characteristic discussion, interdisciplinary dialogues with Cognitive Linguistics and educational sociology, and typological debates) and practical application (covering language teaching, textual analysis, and translation studies). Based on the synthesis, we propose four key directions for future research: expanding the linguistic scope, broadening the population of second language learners, establishing identification criteria for grammatical metaphor in non-English languages, and delineating demetaphorization. The findings of this review offer valuable theoretical references for linguists seeking to refine grammatical metaphor theory, as well as practical guidance for language teachers and curriculum developers aiming to foster learners’ academic literacy. Moreover, the proposed future directions are intended to inspire researchers to explore cross-linguistic and interdisciplinary dimensions, ultimately facilitating a more inclusive and methodologically robust field of grammatical metaphor studies.

## Introduction

Metaphor, initially regarded as a lexical phenomenon and a rhetorical device, is characteristic of the substitution of one word for another to convey a similar meaning. It has a long history in literary studies. It was not until the 1980s, when Lakoff and Johnson (1980) offered a new cognitive interpretation of metaphor, that its study expanded from a lexical-level rhetorical device to clause-level meaning representation, sparking a wave of cognitive metaphor study. Meanwhile, Halliday (2002), the founder of Systemic Functional Linguistics (SFL), proposed the concept of grammatical metaphor, arguing that metaphorical phenomena also exist within grammatical categories, where different grammatical forms can be used to express the same meaning. The proposal of grammatical metaphor represented a significant theoretical innovation within SFL, extending the study of meaning realization from the lexical to the grammatical stratum and providing a powerful framework for explaining abstraction and technicality in academic and scientific discourse.

Over the past four decades, both theoretical and applied research on grammatical metaphor have flourished (e.g., Yan, 2003; Zhang and Lei, 2013; Xuan and Chen, 2019; Qu and Peng, 2020; Li and Guo, 2023). However, existing reviews are often confined to specific sub-types (e.g., ideational grammatical metaphor) or limited to a relatively short time frames. A systematic review that comprehensively and multi-dimensionally presents the full evolutionary landscape—from Halliday’s foundational proposal to the latest interdisciplinary applications and debates—remains to be undertaken.

Therefore, this article aims to review the development of grammatical metaphor within SFL since Halliday’s initial proposal. By employing a methodology of combining systematic retrieval with thematic analysis, we trace the theoretical extensions by other scholars and the applied research that has popularized the theory to outline the developmental trajectory of grammatical metaphor, clarify its current status, identify research gaps, and forecast the development trends in the field. This review seeks to clarify the connotation and denotation of grammatical metaphor research, providing useful insights for related studies.

## Literature review

### Historical background

The history of western grammatical metaphor can be traced back to ancient Greece. The Stoics provided psychological and metaphysical rationales for the “cases” of words and their sub-categories, such as “direct” and “oblique” cases (Alford, 1982). The Middle Ages marked the heyday of grammatical metaphor, where it permeated poetry, sermons, and philosophical treatises. Its simplest form was extended puns, elaborating reality through the double meanings of language itself (Alford, 1982, p. 729). For instance, in 1414, the Duke of Exeter wrote to Henry IV: “Scotland is like an adjective, which cannot exist without a substantive.” Such metaphors, formed from grammatical terms, in turn constructed reality metaphorically. While these early instances were largely confined to rhetorical wordplay, they established the fundamental insight that grammar itself could carry metaphorical meaning (Hu, 1996).

### Theoretical foundations

While these early usages were largely confined to rhetorical wordplay, they provided the historical and conceptual seed for the modern SFL conception of grammatical metaphor. Grounded in linguistic stratification, Halliday’s theory provides a systematic account of how grammar functions as a meaning-making resource. The emergence of grammatical metaphor stems from the stratification of human language. Any language can be viewed as a complex multi-strata semiotic system (Halliday and Matthiessen, 1999), with the semantic stratum and the lexicogrammatical stratum being two crucial layers. The semantic stratum transforms complex human experience into a meaning system, while the lexicogrammatical stratum concretizes the meaning into linguistic expressions. These two strata are linked by realization, that is, certain meaning is realized by specific lexicogrammatical forms.

In the evolution of language, the relationship between these two strata first appeared as a simple one-to-one correspondence, namely the congruent form of realization—the most direct, typical grammatical form of construing experience. For example, a sequence of events is congruently realized by a clause complex, and a process is congruently realized by a verb. However, with the development of human language, this simple correspondence between these two strata began to change, presenting a non-congruent realization, where semantic categories are encoded by atypical grammatical forms. A process, for instance, may be metaphorically realized as a noun (e.g., *arrive* → *arrival*), and a logical relation may be realized as a verb (e.g., *because* → *causes*). It is this changed relationship that constitutes the precondition for the emergence of grammatical metaphor. Halliday and Matthiessen (1999, p. 7) describe grammatical metaphor as “the phenomenon whereby a set of agnate (related) forms is present in the language having different mappings between the semantic and the grammatical categories.”

### Halliday’s theoretical development

Halliday’s research on grammatical metaphor began from the perspective of sociolinguistic variants and was influenced by sociolinguist William Labov and sociologist Basil Bernstein. Halliday (2007) viewed anti-language as a metaphorical variant of everyday language in the early stage and proposed that metaphoricity could be found at different levels of linguistic system, including phonology, grammar, and semantics, which lay the foundation for his subsequent grammatical metaphor study within SFL. Based on chronological and logical analysis of Halliday’s seminal works, his theoretical development of grammatical metaphor can be summarized into three stages (see Table 1).

**Table 1:** Three stages of Halliday’s theoretical development of grammatical metaphor.

Period	Stage	Core theoretical advancement
1980s–1990s	The establishment of the functional perspective	Postulation of the congruent/metaphorical continuum; Primary classification into ideational and interpersonal grammatical metaphor; Linkage to textual mode.
late 1990s - early 21st century	The development of a stratified and evolutionary view	Explanation of congruent forms from the perspective of linguistic evolution; Elaboration of “syndromes of grammatical metaphor”; Explanation of grammatical metaphor’s origin in the tension between strata; Enrichment for semogenesis from the perspective of linguistic logogenesis.
21st century	The integration of transgrammatical semantic domains	Proposal of the concept “transgrammatical semantic domains”; Emphasis on grammatical metaphor’s role in expanding meaning potential; Elevation of the status of interpersonal grammatical metaphor.

The first stage (1980s–1990s) was the establishment of the functional perspective. In the first and second editions of *An Introduction to Functional Grammar* (Halliday, 1985), Halliday systematically expounded the theory of grammatical metaphor. Taking the clause as the unit of analysis, he distinguished between the congruent form and the metaphorical form, which form a continuum. Crucially, Halliday emphasized that choosing a metaphorical form over a congruent one is itself a meaningful choice, adding semantic nuances rather than simply paraphrasing (Halliday, 1985). Halliday (1985) further classified grammatical metaphor into two major types: ideational grammatical metaphor and interpersonal grammatical metaphor. The former is mainly manifested in the transfer of transitivity processes and in “rank-shift” from clause complex to clause to group/phrase, with nominalization identified as its most powerful resource. A classic example is the metaphorical realization of a material process *they arrived at the summit on the fifth day* (the congruent form) by a mental process *the fifth day saw them at the summit* (the metaphorical form). The latter is mainly manifested in the explicit realization of mood and modality meanings. A common example is that the congruent clause *it probably is so* (modal Adjunct) is metaphorically realized as the projecting clause *I think it is so* (explicit subjective modality).

The second stage (late 1990s - early 21st century) was the development of a stratified and evolutionary view. Through a series of studies on scientific and technical texts, Halliday (2004) deepened the theory of grammatical metaphor from the perspectives of linguistic stratification and evolution: ① Replacing metaphorical/congruent forms with “Attic/Doric” and redefining congruent forms from the perspective of linguistic evolution; ② Refining grammatical metaphor into 13 types and referring to them collectively as “syndromes of grammatical metaphor,” emphasizing they are characteristic of “semantic junction.” “Junctional elements will always have two categories in their description” (Halliday and Matthiessen, 1999, p. 244), e.g., in *[poverty] increases* (the congruent form) = > *increasing [poverty]* (the metaphorical form), there is an obvious relationship between *[poverty] increases*= > *increasing [poverty]*, event as quality; ③ Pointing out that grammatical metaphor stems from the tension between the semantic and the lexicogrammatical strata and is a metaphorical extension of semantic system. Meanwhile, Halliday enriched semogenesis from the perspective of logogenesis (Halliday and Matthiessen, 1999).

The third stage (21st century) was the integration of transgrammatical semantic domains. In the third and fourth editions of *An Introduction to Functional Grammar* (Halliday and Matthiessen, 2004, 2014), the theory of grammatical metaphor was further integrated and expanded: ① Proposing the concept of “transgrammatical semantic domains”: the same semantic domain can be realized by multiple different grammatical domains; the reconfiguration of this realization relationship constitutes metaphor. ② Emphasizing the functions of grammatical metaphor in expanding meaning potential and constructing text; ③ Elevating the status of interpersonal grammatical metaphor, arguing that interpersonal projection (e.g., explicit subjective/objective modality) achieves equally significant meaning expansion as ideational grammatical metaphor by absorbing ideational resources.

### Synthesis of existing reviews

Building on Halliday’s three-stage foundational work, several scholars have undertaken reviews of subsequent developments. For instance, Yan (2003) mainly discussed the development of the grammatical metaphor theory by Halliday and others in the past decade (from the early 1990s to the early 2000s) and its theoretical significance. Zhang and Lei (2013) provided an early synthesis of grammatical metaphor in China while Xuan and Chen (2019) offered a synthesis of research on grammatical metaphor in English world. Qu and Peng (2020) analyzed the development and situation of grammatical metaphor research of Chinese scholars between the years of 1996 and 2020 based on CiteSpace analysis tool and CNKI database. More recently, Li and Guo (2023) identified the frontiers and trends of L2 ideational grammatical metaphor. These reviews have significantly advanced our understanding of grammatical metaphor. However, their scope has necessarily been confined to either theoretical development, particular sub-types, specific application domains, shorter time frames, or specific areas. Consequently, a comprehensive, multi-dimensional synthesis that maps the full evolutionary trajectory of grammatical metaphor research—from Halliday’s initial proposal to the latest theoretical debates and interdisciplinary applications—and projects future directions has yet to be conducted. The present review addresses this gap by posing the following research questions:

*Q1*: What is the developmental trajectory of theoretical research on grammatical metaphor advanced by scholars following Halliday?

*Q2*: What is the current status of practical applications of grammatical metaphor in various fields?

*Q3*: What are the future developmental trends and directions for grammatical metaphor research?

## Methodology

### Literature search strategy

To comprehensively trace the development of grammatical metaphor since its proposal by Halliday, a systematic literature search was conducted in two phases.

Phase 1: Foundational search was completed in late 2024. The core body of literature was established in this phase, covering:

① Database search: Primary searches were performed in Web of Science (Core Collection) and CNKI (CSSCI). We used the English terms “grammatical metaphor,” “ideational grammatical metaphor,” “interpersonal grammatical metaphor” or “nominalization” to search titles, abstracts and keywords in Web of Science. We used the Chinese terms “语法隐喻,” “概念语法隐喻,” “人际语法隐喻” or “名物化” to search titles, abstracts and keywords in CNKI.

② Supplementary search: To ensure the coverage of foundational and high-impact works, we also traced the publication list of key scholars (e.g., M. A. K. Halliday, C. M. I. M. Matthiessen, J. R. Martin, 杨炳均, 杨延宁) and the reference lists of relevant review articles and major publications using the snowballing technique.

Phase 2: Final update search was conducted on 16 December 2025. To ensure the review’s timeliness prior to submission, a targeted update search was performed. This phase focused specifically on retrieving new and Online First publications in 2025 using the same databases and strategies.

### Inclusion and exclusion criteria

The selection of literature adheres to the following criteria to ensure the review remained focused on the theoretical lineage within SFL.

#### Inclusion criteria

1. Studies published or made formally available online from 1985 (Halliday’s first proposal) to 16 December 2025.

2. The core discussion of the studies must center around grammatical metaphor within the framework of SFL.

3. Peer-reviewed journal papers, academic monographs, book chapters and seminal early works (e.g., by Halliday and Matthiessen).

4. Studies published in English or Chinese.

#### Exclusion criteria

1. Studies published in languages other than English or Chinese. As we could not perform an exhaustive search on non-English and non-Chinese publications, we chose to exclude these studies.
2. Studies primarily focused on metaphor in traditional rhetoric or cognitive linguistics, without substantive engagement with the SFL concept of grammatical metaphor.
3. Unpublished studies presented as conference papers, theses and dissertations.
4. Studies whose full text was not accessible through institutional or reasonable request channels.

### Synthesis procedure

Given the theoretical density and evolving nature of grammatical metaphor concept, a transparent and structured approach to synthesis is essential for tracing its development. The following qualitative procedure was adopted:

1. In-depth analysis of the foundational works: We began with a close reading and critical analysis of the seminal works by Halliday and his key co-authors to trace the evolution of their ideas and to identify core concepts and turning points in the development of grammatical metaphor according to the chronological order and theoretical logic.
2. Thematic categorization of the expanded literature: For the body of expanded literature beyond the foundational core, we employed a thematic analysis approach. This involved a careful reading and comparison of studies to identify each study’s primary focus. Our analysis proceeded in two stages: First, the studies were classified into two major streams based on their focus on either theoretical development or practical application. Subsequently, within each major stream, specific prevalent sub-themes were identified through continued thematic analysis (e.g., debates on typology within theoretical development, application in translation within practical application). Through this process, we developed and applied the following coding categories for each study: (1) author(s), (2) publication year, (3) title, (4) source, (5) major stream, and (6) sub-themes.
3. Formulation of future research directions: Based on the thematic synthesis of both foundational and expanded literature, we found unresolved debates and emerging trends. This allowed us to infer directions for further study, thereby moving from a descriptive review to a prospective agenda for the field.

After applying the inclusion and exclusion criteria, a total of 293 studies were retained for analysis. Following the thematic synthesis procedure, among these studies 15 were Halliday’s own works on grammatical metaphor, 141 focused on theoretical development, and 137 on practical application. For a complete list of all 293 studies included in this review, see Supplementary Appendix A.

## Findings

### Research question 1: what is the developmental trajectory of theoretical research on grammatical metaphor advanced by scholars following Halliday?

The 278 studies (141 theoretical + 137 applied) constitute the primary data for our thematic analysis. Through the thematic synthesis analysis of these studies, we have identified predominant sub-themes within two major streams of theoretical development and practical application, the distribution of which is summarized in Table 2. Regarding the first research question concerning the theoretical development of grammatical metaphor beyond Halliday’s own work, the thematic analysis of 141 studies reveals the following three predominant sub-themes.

**Table 2:** Distribution of developments in grammatical metaphor study by other scholars.

Major streams	Sub-themes	Studies	Percentage
Theoretical development	semantic and characteristic discussion	46	33%
	interdisciplinary studies	40	28%
	types of grammatical metaphor	26	18%
	theoretical introduction and interpretation	22	16%
	others	7	5%
Practical application	language teaching	59	43%
	textual analysis	53	39%
	translation studies	20	15%
	others	5	3%

#### Semantic and characteristic discussion

Accounting for 33% of the studies on theoretical development, the discussions on the semantics and characteristics of grammatical metaphor forms a substantial research focus. It is generally agreed that grammatical metaphor involves semantic compounding; that is, the congruent form realizes the selection of single semantic feature, while the metaphorical form realizes the selection of compound semantic features (Ravelli, 2003; Derewianka, 2003; He, 2008). This is not only a method for constructing new meanings but also expresses more nuanced semantic distinctions (Jiang, 2014). Its generation mechanism can be interpreted from the perspectives of cognition and context (Fan, 2007; Lin and Yang, 2010). The studies have also revealed several systematic characteristics of grammatical metaphor. For example, Ravelli (1985) earlier noted that ideational grammatical metaphor has the characteristics of paradigmatic recursion and clusters, which are similar to “syndromes of grammatical metaphor” and the “domino effect” proposed by Halliday and others later. Furthermore, empirical studies have shown that the use of interpersonal grammatical metaphor is influenced by topics of conversation and social relationships between interlocutors (e.g., Yang, 2013; He and Yang, 2018).

#### Interdisciplinary discussion

Accounting for 28% of the studies on theoretical development, the interdisciplinary studies has been a major drive for broadening the horizons of grammatical metaphor research, mainly unfolding along two paths: dialogue with Cognitive Linguistics and integration with educational sociology.

Firstly, the majority of interdisciplinary studies (about 54%) focus on the dialogue with Cognitive Linguistics. Halliday had already considered cognitive perspective in his early studies and placed cognition within a subcategory of mental processes (Halliday and Matthiessen, 2004). This makes grammatical metaphor regarded as a cognitive construct or a mechanism beyond the language system. Scholars actively apply the theories of Cognitive Linguistics to explain ideational grammatical metaphor, e.g., using theories such as construction and image schema to interpret the cognitive basis of grammatical metaphor (He, 2016; Zhao, 2018). Meanwhile, scholars have paid increasing attention to the scientization of methodology. In response to the issue of subjectivity in traditional analyses, Fontaine et al. (2025) attempted to introduce the category of “aspect” to provide more stable and precise operational criteria for the identification of nominalization, which represents the important advancement of methodology.

Secondly, a large number of interdisciplinary studies (about 24%) explore the integration with educational sociology. The dialogue between SFL and educational sociology has a long history. Halliday incorporated the spirit of Bernstein’s code theory into pedagogical practice. The Sydney School, represented by Martin, further integrated ideational grammatical metaphor with Bernstein’s theory of knowledge structure and Maton’s Legitimation Code Theory to explain the cumulative construction of knowledge. In recent years, ideational grammatical metaphor has been not only viewed as a key linguistic resource for realizing meaning packing/unpacking of knowledge and regulating semantic density/gravity (Martin, 2013), but also deep understood through dynamic models. For example, Wu (2024) introduced semantic wave theory and depicted the dynamic advancement progress of nominalization in constructing hierarchical knowledge structures. Miao (2025) constructed an analytical framework centered on logical grammatical metaphor and empirically revealed its specific trajectory in driving the abstraction of disciplinary knowledge.

#### Discussion on the types of grammatical metaphor

The discussion on types of grammatical metaphor accounts for 18% of the studies on theoretical development. The core debate within this sub-theme revolves around whether the independent type of textual metaphor should be established. Martin (1992, p. 416) explicitly proposed that textual metaphor, like ideational and interpersonal grammatical metaphor, should fall within the scope of grammatical metaphor study and be the third type. Scholars such as Thompson (2014, p. 251) support this view. However, Halliday did not adopt it, and Yang (2018) also questioned the definition of textual metaphor. In recent years, the discussion on the type of grammatical metaphor has shown a trend toward refinement and integration. For instance, Yang and Gao (2023) conducted an in-depth exploration of polarity metaphor, a type of grammatical metaphor which has both ideational and interpersonal meanings. Lai and Su (2025) revealed how interpersonal grammatical metaphor iconically constructs distant social power relations through grammatical complexity from the dimension of interpersonal rhetorical effects. Lin (2025) linked grammatical metaphor with cognitive mechanisms of metaphor and metonymy by proposing a dominant “metaphor” theory and attempted to construct a higher-level theoretical framework.

### Research question 2: what is the current status of practical applications of grammatical metaphor in various fields?

In response to the second research question regarding the practical application, this review finds that applied research on grammatical metaphor has shown a trend of diversification, with studies primarily focusing on three major fields. As shown in Table 2, these three fields collectively account for 97% of all application studies. Table 3 provides a qualitative overview of each field’s core focus and key findings.

**Table 3:** Overview of three major applied fields of grammatical metaphor research.

Research fields	Core focus	Key findings and progress
Language teaching	L1/L2 acquisition rules and teaching strategies	L1 children start using grammatical metaphor early; L2 beginner learners have already developed preliminary grammatical metaphor ability; L2 advanced learners often show insufficient/uneven grammatical metaphor use; grammatical metaphor proficiency correlates with writing maturity.
Textual analysis	Ideology and knowledge construction of various formal texts	Grammatical metaphor is a typical feature of formal texts and used to analyze various formal texts such as news, economic, political, and academic discourse; application is significantly higher in science than arts.
Translation studies	Translation strategies, cross-linguistic comparison, and cognitive processes	Evolved from strategies discussion (e.g., unpacking) and cross-linguistic comparisons to empirical investigations of translators’ cognitive processes.

#### Language teaching

Grammatical metaphor has been widely applied in the field of language teaching (about 43% of applied research). Researchers within this sub-theme focus on acquisition rules (L1 and L2 acquisition) and teaching strategies of grammatical metaphor. In first language acquisition (L1 acquisition), follow-up studies have shown that children start to use metaphorical expressions at an earlier age (Torr and Simpson, 2003), but the acquisition of different types of grammatical metaphor is not consistent (Derewianka, 2003), and the development of grammatical metaphor proficiency is positively correlated with writing proficiency (Liardét, 2016). In second language acquisition (L2 acquisition), studies indicate that learners have already developed preliminary grammatical metaphor ability in middle school or even upper grades of primary school. The extent to which this ability is used is an important indicator for measuring complexity and maturity of second language (L2) (especially academic) writing (e.g., Ryshina-Pankova, 2015). However, compared with native speakers, advanced learners still encounter issues such as insufficient usage or uneven distribution (e.g., Chen and He, 2024; Dong et al., 2024; Guo and Li, 2025). To promote teaching, scholars have explored various approaches such as “meaning-based writing” instructional design and Production-Oriented Approach. The latest development is reflected in the design of teaching and research tools: based on a large academic corpus, McGrath and Liardét (2025) developed *A Grammatical Metaphor Word List*, providing a valuable resource tool for large-scale assessment of learner development and design of focused instructional activities.

#### Textual analysis

Grammatical metaphor has been utilized in the field of textual analysis (about 39% of applied research). Due to its functions in experiential representation and construction of objectivity, grammatical metaphor is considered a typical feature of formal adult text and an important tool for analyzing ideology and knowledge construction of various formal texts (Halliday, 2002). Studies not only cover traditional news discourse (e.g., headline ambiguity, responsibility construction), economic discourse (e.g., stylistic function of business letters), political discourse (e.g., power and ideology analysis), and academic discourse (including multiple disciplines such as history, medicine, chemistry, and mathematics), but also extend to new media discourse in the digital age. For example, Zhang and Yang (2025), combining mood metaphor with Bourdieu’s theory of symbolic violence, analyzed how video game discourse exerts hidden symbolic power on players through metaphorical interrogatives. Quantitative research further reveals disciplinary differences in the use of grammatical metaphor, confirming that its application in science discourse is significantly higher than that in the arts (Zhu and Xu, 2024).

#### Translation studies

The application of grammatical metaphor has been explored in translation studies (about 15% of applied research). Research in this field has evolved from initial discussions on translation strategies and cross-linguistic comparisons to empirical investigations of translators’ cognitive processes. For instance, early studies explored how to apply “unpacking” strategy to handle grammatical metaphor in translation (Steiner, 2002a), how to utilize the power of grammatical metaphor to convey ideology (Zhu and Zhang, 2015), and explored similarities and differences of grammatical metaphor between languages such as English-Chinese and English-German (e.g., Halliday, 1993; Steiner, 2002b). Recently, through experimental research, Yang et al. (2025) found that the extent to which metaphor is used in the source text and the translator’s professional level have significant influence on time allocation in translation and the maintenance of metaphor in the target text. This reveals the complex interaction among grammatical metaphor, translation expertise and translation performance and pushes translation process research toward a more refined cognitive-empirical level.

### Research question 3: what are the future developmental trends and directions for grammatical metaphor research?

Regarding the third research question on future developmental trends, the thematic synthesis of the 293 studies reveals that while the field has yielded fruitful results, there remains significant room for development. We propose four key directions for future research.

#### Direction 1: expanding the linguistic scope of grammatical metaphor research

Our synthesis reveals that English has been the primary object of analysis for grammatical metaphor studies, while systematic research on grammatical metaphor in other languages is lacking. From the perspective of linguistic universals, grammatical metaphor, arising from the interaction between the semantic and grammatical strata (Halliday and Matthiessen, 1999), should manifest universally, albeit with language-specific characteristics. Recent pioneering work on Chinese (Yang, 2020) and Spanish (Salum, 2024) demonstrates that non-English languages exhibit distinct grammatical metaphor patterns tied to their typological features. For instance, Yang (2020) systemic analysis of Chinese reveals that topic-prominence structures generate unique metaphorical realizations absent in English. Future systematic typological research on grammatical metaphor should be conducted to enrich grammatical metaphor theory.

#### Direction 2: broadening the L2 learner population in grammatical metaphor research

According to the review of L2 acquisition research, current L2 grammatical metaphor studies primarily focus on undergraduate or graduate, with less attention paid to primary and secondary school learners. Although grammatical metaphor is considered a feature of adult language (Halliday and Matthiessen, 2004), the phenomenon of native language grammatical metaphor has already emerged in preschool children’s language. Regarding L2, the phenomenon of L2 grammatical metaphor has already appeared in upper-grade primary school learners’ language (Lim and Kellogg, 2008). The pedagogical urgency is underscored by curriculum reforms such as *Compulsory Education English Curriculum Standards* (2022 Edition), which integrate scientific and technical discourse from upper-grade primary to secondary levels and emphasize interdisciplinary problem-solving and comprehension of argumentative text. These requirements, to some extent, imply the need for cultivating grammatical metaphor competence. Future research should extend the population from university students to primary and secondary school learners, distinguishing natural development from explicit instruction effects, and develop age-appropriate pedagogical frameworks.

#### Direction 3: establishing identification criteria for non-English grammatical metaphor

The identification of grammatical metaphor relies on a complete systemic functional analytical framework. However, the existing known functional analyses are almost established based on English language facts (Yang, 2020, p. 74). This causes a circular issue: If we use criteria derived from English to analyze other languages, we risk either misidentifying grammatical metaphor or missing the real phenomena. Future research should focus on creating analytical frameworks for grammatical metaphor in non-English languages. Meanwhile, it is necessary to conduct in-depth exploration of the special linguistic phenomena in the language to avoid unnecessary ambiguous areas when identifying grammatical metaphor, so as to enhance the scientificity and accuracy of grammatical metaphor identification, thereby meaningful cross-linguistic comparisons can be made.

#### Direction 4: delineating demetaphorization in grammatical metaphor

Halliday (1985, p. 348), in explaining ideational grammatical metaphor, noted that there is no clear boundary between congruent and metaphorical forms, and the history of language is a history of demetaphorization. Similar to lexical metaphor, initially metaphorical expressions can gradually conventionalize: for example, in expressions like “have a bath” and “make a mistake,” processes are encoded as nominal groups. Although the expression forms are non-congruent, they have become part of the English system and serve as the unmarked form for encoding these process types. This raises some questions: At what point does a grammatical metaphor lose its metaphorical status, akin to “dead metaphors” in lexical metaphor (Jiang, 2014)? How do “dormant” and “active” grammatical metaphors influence language change today? Could the process of demetaphorization speed up in digital times? Future research needs to develop diachronic corpus methodologies to track demetaphorization trajectories and to better understand the continuum between congruent and metaphorical forms.

## Discussion

The findings presented above offer a comprehensive map of grammatical metaphor research over the past four decades. In this section, we will discuss these findings by focusing on two core themes: theoretical developments and applied research.

### Theoretical developments

Our synthesis reveals that semantic and characteristic discussions constitute the largest proportion of theoretical studies (33%), indicating that scholars have consistently engaged with the semantic complexity of grammatical metaphor, particularly its semantic compounding nature. This focus has been established in early work (e.g., Ravelli, 2003) and aligns with Halliday’s emphasis on meaning potential. This suggests that understanding how grammatical metaphor condenses and repackages meanings has been a concern. While previous reviews have noted this theoretical focus—whether as “language description” in the English-language literature (Xuan and Chen, 2019, p. 222), as “theory refinement” in the Chinese context (Zhang and Lei, 2013, p. 3), or as theoretical re-interpretation of Halliday’s evolving model (Yan, 2003), our synthesis provides a long-term quantitative result of analysis, indicating that it has maintained a significant influence over the past four decades.

The substantial proportion of interdisciplinary studies (28%) reflects a healthy dialogue between SFL and other disciplines, notably Cognitive Linguistics and educational sociology. These studies have unfolded along two distinct paths. The cognitive path, exemplified by He (2016) and Zhao (2018), addresses the question of “how is grammatical metaphor possible.” The sociological path, represented by Martin (2013) and Wu (2024), addresses the question of “what does grammatical metaphor do.” This finding extends earlier observations by Zhang and Lei (2013), who noted the emergence of interdisciplinary work in the Chinese context, and Qu and Peng (2020), who identified cognitive approaches as a growing trend. Our synthesis indicates that such dialogues have now become a major direction of theoretical development, positioning grammatical metaphor as a bridge between linguistic description and broader cognitive and social theories.

The persistent debate on grammatical metaphor typology, accounting for 18% of theoretical studies, highlights a theoretical tension that remains unresolved. Regarding the definition of textual metaphor, scholars still have opinions: Martin (1992) advocated classifying it as the third type, while Halliday and Matthiessen (2004, 2014) never formally adopted this expansion. And Yang (2018) offered a systematic critique, arguing that the phenomena classified as textual metaphor are often merely textual effects rather than an independent domain of grammatical metaphor. Similarly, the identification and classification of interpersonal grammatical metaphor have always been difficult to clearly define. He and Wen (2017) pointed out that their boundaries are vague, while Taverniers (2018) traced their historical evolution to reveal their constantly changing. This debate, largely absent from earlier reviews (e.g., Yan, 2003), has gained significant attention in later academic research and has become an emerging point of theoretical exploration (Xuan and Chen, 2019). The persistence of these debates suggests that the attention in this field has been focused on the precision of classification. Recent studies have begun to move beyond this classification deadlock. For instance, Lin (2025) “trope” theory, Yang and Gao (2023) polarity metaphor, and Lai and Su (2025) analysis of interpersonal metaphor in literary interviews. These comprehensive efforts point toward a reconceptualization of grammatical metaphor, no longer viewing them as a fixed list of types, but rather as a dynamic resource operating across metafunctions.

### Applied research

In the application fields, the dominance of language teaching (43%) and textual analysis (39%) highlights the theory’s practical value. This proportion is highly consistent with Xuan and Chen (2019) findings, in which education constituted the largest category (33%), indicating that the application of grammatical metaphor theory in education has formed a stable trend. The consistent finding is that even highly proficient L2 learners still encounter difficulties in understanding grammatical metaphor (e.g., Chen and He, 2024; Dong et al., 2024; Guo and Li, 2025). This finding highlights the pedagogical challenges from this linguistic resource. And our synthesis confirms its cross-linguistic and cross-contextual pervasiveness.

However, our synthesis also reveals two limitations in educational applications. Firstly, research participants are mainly university students, while evidence from basic education is relatively scarce. This research phenomenon is related to the historical development of grammatical metaphor theory, which initially aimed to explain abstraction and technicality in scientific discourse (Halliday, 1993), and scientific discourse is considered a feature of adult language, leading early research to focus on higher education. Yet subsequent studies have shown that grammatical metaphor emerges much earlier in children’s language development (Torr and Simpson, 2003; Lim and Kellogg, 2008), suggesting that the developmental trajectory of grammatical metaphor competence is a continuum from “sprout” to “maturity.”

Secondly, research remains primarily descriptive. Most studies remain at the diagnostic level—revealing problems such as learners’ insufficient use and uneven distribution (e.g., Chen and He, 2024; Dong et al., 2024; Guo and Li, 2025)—but few have transformed these diagnostic results into systematic pedagogical interventions. The transformation of grammatical metaphor as a pedagogical framework is still in its infancy, while its utility as an analytical framework has been fully proven. McGrath and Liardét (2025) *Grammatical Metaphor Word List,* based on a large academic corpus, marks an important shift from problem-describing to problem-solving, providing a tool for large-scale assessment and targeted instruction.

The expansion of textual analysis to new media discourse demonstrates the theory’s adaptability to evolving communicative practices. While Xuan and Chen (2019) mapped applications to traditional text types, our synthesis reveals an emerging extension to digital genres—a direction not captured in previous reviews. This extension suggests that grammatical metaphor analysis can reveal the construction of power relations and ideologies in the new media communication contexts.

Translation studies, though smaller in volume (15%), are evolving from strategy-based discussions toward cognitive-process research. Early studies explored translation strategies and cross-linguistic comparisons (Steiner, 2002a, 2002b; Zhu and Zhang, 2015); more recent studies, such as Yang et al. (2025), employs experimental methods to investigate the interaction between translator’s expertise and metaphoricity of source-text. This methodological shift from strategy discussion to empirical research represents a significant advance beyond the translation research surveyed in earlier reviews (e.g., Zhang and Lei, 2013).

## Conclusion

This article has systematically reviewed the historical development and current status of grammatical metaphor research within SFL over the past four decades. Through a structured methodology—including database search, snowballing technique, and thematic synthesis—we have traced the trajectory of Halliday’s three-stage theoretical development and examined the subsequent theoretical expansions and practical applications by other scholars.

Based on the synthesis, our review reaffirms that grammatical metaphor remains a vital theoretical construct, particularly in interpreting the abstraction and technicality in academic and scientific discourse. The characteristic of this theoretical field lies in its continuous exploration of semantic complexity, strong interdisciplinary dialogue, and ongoing typological debates. Applied research has demonstrated the practicality of this theory in areas such as language teaching, textual analysis, and translation studies, with educational applications constituting the most active field.

The review also points out several directions for future research. Theoretically, this field should move from the debates on classification toward functional integration, investigating the interaction patterns of different metaphorical dimensions in discourse, and should pay attention to the phenomenon of demetaphorization that has not been sufficiently studied. Methodologically, future research should incorporate more quantitative and mixed approaches, and be combined with qualitative analysis. In the meanwhile, more reliable identification criteria should be developed, especially for non-English languages, to enhance cross-study comparability and empirical rigor. Pedagogically, research should extend to primary and secondary learners, and should transform diagnostic findings into accessible teaching tools for frontline educators. In applied domains, the expansion toward professional communication and digital genres is expected to test and enrich this theory. It is hoped that this work not only consolidates current understandings but also encourages further systematic inquiries, thereby promoting grammatical metaphor research toward greater linguistic inclusiveness, methodological soundness, and pedagogical relevance.

Finally, it must be acknowledged that this review is primarily based on literature in English and Chinese. Incorporating literature in other languages will enable future research to achieve a more comprehensive global perspective on grammatical metaphor.
