# Supplementary material for: Grammatical metaphor studies: historical review and outlook
Source: Front Psychol. 2026 Mar 27;17:1774523. doi: 10.3389/fpsyg.2026.1774523 (PMC13066154; doi:10.3389/fpsyg.2026.1774523)
Supplement: Supplementary file 1 [file Supplementary_file_1.pdf]

Appendix A

Table A: The studies within theoretical development

No.	Author(s)	Year	Title	Source	Sub-theme
1	Arús, J	2003	Ambiguity in grammatical metaphor: One more reason why the distinction transitive/ergative pays off	*Grammatical Metaphor: Views From Systemic Functional Linguistics*	Semantic and Characteristic
2	Asare, E., Mwinlaaru, I. N., and Ofori, E. A.	2024	Construing joy as body parts in Akan: Synergizing conceptual metaphor and transitivity Analyses	*Word-Journal of the International Linguistic Association*	Interdiscipline
3	Chang, C. G.	2004	The reconstruction of grammatical metaphor and experience	*Foreign Language Teaching and Research*	Semantic and Characteristic
4	Chen, C. M., and He, Q. S.	2024	A corpus-Based study of metaphor of modalization in English academic writing	*Sage Open*	Semantic and Characteristic
5	Chen, S. K.	2018	Rank shift and compound prepositions: A functional grammar analysis of projected prepositional phrases in Chinese	*Journal of Xi'an International Studies University*	Semantic and Characteristic
6	Chen, S. X., and Stepanov, I.	2024	Trends in mutual transpositions of lexical and grammatical noun categories in Russian language	*Sage Open*	Semantic and Characteristic
7	Chen, X. R.	2014	A pragma-cognitive account of grammatical metaphor	*Journal of Foreign Languages*	Interdiscipline
8	Cong, Y. X.	2011	On the restraint and expansion of ideational grammatical metaphor	*Journal of Foreign Languages*	Semantic and Characteristic
9	Cong, Y. X.	2014	The contributions and issues of grammatical metaphor theory of Systemic Functional Linguistic	*Journal of PLA University of Foreign Languages*	Theoretical interpretation
10	Cong, Y. X., and Wang, H. Y.	2013	Recategorization of ideational grammatical metaphors based on semantic change	*Modern Foreign Languages*	Semantic and Characteristic
11	Cong, Y. X., and Wang, H. Y.	2017	The cognitive features and explanation of congruence in grammatical metaphor	*Foreign Languages in China*	Interdiscipline
12	Dai, W. P., and Gao, L. J.	2009	Grammatical metaphor: Evidentiality and contribution	*Lanzhou Academic Journal*	Theoretical interpretation
13	Dong, J.	2021	Experiential semantic change in quality-thing grammatical metaphor	*Foreign Language Research*	Type
14	Dong, J.	2021	*Semantic Change in Grammatical Metaphor*	China Ocean University Press	Semantic and Characteristic
15	Dong, J., and Zhang, D. L.	2017	Reflection on the theory of grammatical metaphor: Exploring textual metaphor	*Modern Foreign Languages*	Type
16	Dong, M.	2018	Cross-linguistic logical grammatical metaphor in relation to inter-Lingual explicitation	*Journal of PLA University of Foreign Languages*	Semantic and Characteristic

17	Dong, M., and Fang, A. C. Y.	2021	Shell nouns as grammatical metaphor revealing disparate construals: Investigating the differences between British-English and China-English based on a comparable corpus	*Corpus Linguistics and Linguistic Theory*	Type
18	Dong, M., Fang, A. C., and Qiu, X. X.	2020	Shell nouns as grammatical metaphor in knowledge construal: Variation across science and engineering discourse	*Lingua*	Type
19	Dong, M., and Xu, L. Y.	2017	Logical grammatical metaphor: A local grammar perspective	*Foreign Languages in China*	others
20	Fan, W. F.	2000	Metaphors of mood in English	*Journal of Foreign Languages*	Type
21	Fan, W. F.	2007	A comprehensive pattern of grammatical metaphor	*Foreign Language Education*	Semantic and Characteristic
22	Fang, Y. G., and Cong, Y. X.	2020	A cognitive and semantic study of denominal adjectivisation in English and Chinese — A grammatical metaphor perspective	*Journal of PLA University of Foreign Languages*	Interdiscipline
23	Ferreira, A. A.	2020	Sociocultural development in the spectrum of concrete and abstract ideation	*Mind Culture and Activity*	Interdiscipline
24	Fontaine, L., Taverniers, M., Carr, A., and Neumann, S.	2025	Operationalizing grammatical metaphor	*Folia Linguistica*	Type
25	Guarddon-Anelo, M. D.	2011	The role of metonymy and metaphor in grammaticalization: The expression of aspect	*Australian Journal of Linguistics*	Interdiscipline
26	Halliday, M. A. K., and Williams, G.	2023	Exploring the “language” part of language education	*Language Context and Text-The Social Semiotics Forum*	Interdiscipline
27	Hao, J.	2018	Reconsidering “cause inside the clause” in scientific discourse — from a discourse semantic perspective in systemic functional linguistics	*Text & Talk*	Semantic and Characteristic
28	Hao, J.	2020	Nominalisations in scientific English: A tristratal perspective	*Functions of Language*	Semantic and Characteristic
29	Hao, J., and Wang, P.	2022	Chinese nominal groups: The metaphorical realization of figures	*Word-Journal of the International Linguistic Association*	Semantic and Characteristic
30	He, Q. S.	2013	Rethinking transfer dimensions of grammatical metaphor	*Journal of Northeastern University (Social Science)*	Semantic and Characteristic
31	He, Q. S.	2021	A corpus-based study of interpersonal metaphors of modality in English	*Studla Neophilologica*	Type
32	He, Q. S.	2021	A corpus-based study of textual Metaphor in English academic writing	*Studla Linguistica*	Type
33	He, Q. S., and Guo, M.	2021	A corpus-based study of adjectivizations in English	*Lingua*	Semantic and Characteristic

34	He, Q. S., and Wen, B. L.	2017	A corpus-based study of textual metaphor in English	*Australian Journal of Linguistics*	Type
35	He, Q. S., and Yang, B. J.	2014	A study of transfer directions in grammatical metaphor	*Australian Journal of Linguistics*	Semantic and Characteristic
36	He, Q. S., and Yang, B. J.	2018	A corpus-based study of the correlation between text technicality and ideational metaphor in English	*Lingua*	Semantic and Characteristic
37	He, Q. S., and Yang, B. J., and Wen, B. L.	2015	Textual metaphor from the perspective of relator	*Australian Journal of Linguistics*	Type
38	He, Q. S., and Zhang, Q. Q.	2024	A corpus-based study of live grammatical metaphor in English academic writing	*Studla Neophilologica*	Semantic and Characteristic
39	He, W.	2008	Grammatical metaphor： Variation in both form and meaning	*Journal of PLA University of Foreign Languages*	Semantic and Characteristic
40	He, W.	2008	Modal uses of tense: grammatical metaphor	*Foreign Languages and Their Teaching*	Semantic and Characteristic
41	He, Y. J., and Wang, L. L.	2007	On nominal structures in Chinese	*Chinese Language Learning*	Semantic and Characteristic
42	He, Z. Q.	2014	The development and problems in grammatical metaphor theory	*Journal of University of Science and Technology Beijing (Social Sciences Edition)*	others
43	He, Z. Q.	2016	A construction approach to metaphor studies	*Foreign Language Research*	Interdiscipline
44	He, Z. Q.	2024	Modality in SFL revisited: Establishing evidentiality in the System of Modality	*Word-Journal of the International Linguistic Association*	Semantic and Characteristic
45	Holme, R.	2003	Grammatical metaphor as a cognitive construct	*Grammatical Metaphor: Views from Systemic Functional Linguistic*	Interdiscipline
46	Hou, J. B.	2008	From semantic stratum to lexicogrammatical stratum	*Foreign Languages and Their Teaching*	others
47	Hou, J. B.	2008	Grammatical metaphor: New idea and reflection	*Foreign Language Education*	others
48	Hu, Z. L.	1996	Grammatical metaphor	*Foreign Language Teaching and Research*	Theoretical interpretation
49	Hu, Z. L.	2000	Evaluating Halliday's model of grammatical metaphor	*Foreign Language Teaching and Research (bimonthly)*	Theoretical interpretation
50	Hu, Z. L., Zhu, Y. S., Zhang, D. L., and Li, Z. Z.	2005	*An Introduction to Systemic Functional Linguistics*	Peking University Press	Theoretical interpretation
51	Jiang, W. Q.	2014	Grammatical metaphor: Its origin, stages of development, and nature	*Journal of PLA University of Foreign Languages*	Theoretical interpretation
52	Jin, N. N., and Chen, Z. L.	2004	Cognitive effects of grammatical metaphor	*Foreign Language Teaching and Research*	Interdiscipline

53	Lei, C. Y.	2019	New taxonomic proposal for Chinese phraseology	*Circulo De Linguistica Aplicada A La Comunicacion*	Type
54	Li, M.	2010	On the transformation of congruent form to metaphorical form and information density in English sentences	*Journal of Inner Mongolia University (Philosophy and Social Sciences)*	Interdiscipline
55	Li, X. J.	2016	Grammatical metaphor in the scale of text	*Foreign Language Research*	Type
56	Li, W., and Guo, J. H.	2023	Ideational grammatical metaphor in second language studies: Frontiers and trends	*Modern Foreign Languages*	others
57	Li, W., and Yang, B. J.	2024	Towards a system of principles for identifying nominalizing metaphors	*Lingua*	Type
58	Liardét, CL	2016	Grammatical metaphor: Distinguishing success	*Journal of English for Academic Purposes*	Type
59	Lien, C. F.	2011	Development of directionals in southern Min	*Language and Linguistics*	Interdiscipline
60	Lin, Z. J.	2025	Trope: Metaphor, metonymy and grammatical metaphor	*Foreign Languages in China*	Interdiscipline
61	Lin, Z. J., and Dong, X. M.	2017	The metonymic attribute of grammatical metaphor	*Journal of Northeast Normal University (Philosophy and Social Sciences Edition)*	Interdiscipline
62	Lin, Z. J., and Wang, K. F.	2012	A probe into cross-linguistic grammatical metaphor	*Foreign Language Research*	Interdiscipline
63	Lin, Z. J., and Yang, Z.	2010	A study of semantic relations and rankshift directions in grammatical metaphor	*Foreign Language Teaching and Research*	Interdiscipline
64	Lin, Z. J., and Yang, Z.	2016	On the pragmatic motivation of grammatical metaphor	*Modern Foreign Languages*	Interdiscipline
65	Lin, Z. J., and Zhang, H.	2022	The embodiment basis of ideational grammatical metaphor	*Foreign Languages in China*	Interdiscipline
66	Lin, Z. J., and Zhang, S. Y.	2018	On the semogenic motivations of grammatical metaphor	*Foreign Languages and Their Teaching*	Semantic and Characteristic
67	Liu, C. Y.	2003	The stylistic value of grammatical metaphor	*Modern Foreign Languages*	Semantic and Characteristic
68	Liu, C. Y.	2005	The reverse transfer of rank-shift of ideational and interpersonal metaphor	*Foreign Language Teaching and Research*	Semantic and Characteristic
69	Liu, C. Y.	2008	*Functional-cognitive Stylistic Approach to Grammatical Metaphor*	Xiamen University Press	Interdiscipline
70	Liu, T. T., and Zhang, Y.	2014	Ideational metaphor from a cognitive perspective	*Modern Foreign Languages*	Interdiscipline
71	Liu, Y. H.	2001	On congruence	*Journal of Foreign Languages*	Semantic and Characteristic
72	Luo, Z. B.	2016	On the semantic wave of configuring grammatical metaphor	*Journal of Xi'an International Studies University*	Interdiscipline
73	Luo, Z. B., and Jiang, Y. H.	2015	The construction model of semantic wave of grammatical metaphor	*Foreign Languages Research*	Interdiscipline

74	Ma, Y. L., and Tao, M. Z.	2007	Grammatical metaphor, construction and analogical mapping	*Foreign Language Education*	Interdiscipline
75	Martin, J. R.	1992	*English Text: System and Structure*	John Benjamins Publishing Company	Type
76	Martin, J. R.	2008	Incongruent and proud: De-vilifying "nominalization"	*Discourse & Society*	Semantic and Characteristic
77	Martin, J. R., Gao, Y. M., Li, H. B., Song, C. F., and Wei, M. L.	2021	Martin on discourse semantics, genre, educational linguistics	*Language Context and Text- The Social Semiotics*	Interdiscipline
78	O'Halloran, K. L.	2008	Systemic functional-multimodal discourse analysis (SF-MDA): Constructing ideational meaning using language and visual imagery	*Visual Communication*	Interdiscipline
79	Pecman, M.	2014	Variation as a cognitive device How scientists construct knowledge through term formation	*Terminology*	Interdiscipline
80	Pu, Z. F.	2015	Semantic changes from the perspectives of lexical metaphor and grammatical metaphor	*Journal of the Chinese Society of Education*	Semantic and Characteristic
81	Qian, K.	2017	Recategorization of grammatical metaphor based on contrastive studies of English and Chinese	*Journal of Xi'an International Studies University*	Type
82	Qu, Y. M.	2014	*Study on Grammatical Metaphor*	Northeast Normal University Press	Theoretical interpretation
83	Ravelli, L. J.	1985	*Metaphor, Mode and Complexity: An Exploration of Co-varying Patterns*	University of Sydney	Semantic and Characteristic
84	Ravelli, L. J.	1988	Grammatical metaphor: An initial analysis	*Pragmatics, Discourse and Text: Some Systemically-Inspired Approaches*	Theoretical interpretation
85	Ravelli, L. J.	2003	*Grammatical Metaphor: Views From Systemic Functional Linguistic*	John Benjamins	Theoretical interpretation
86	Ravelli, L. J.	2003	Renewal of connection: integrating theory and practice in an understanding of grammatical metaphor	*Grammatical Metaphor: Views From Systemic Functional Linguistics*	Theoretical interpretation
87	Ritchie, L. D., and Zhu, M.	2015	Nixon stonewalled the investigation: Potential contributions of grammatical metaphor to conceptual metaphor theory and analysis	*Metaphor and Symbol*	others
88	Salum, N. L.	2024	The use of logical metaphors to construct causality in Spanish	*Logos-Revista De Linguistica Filosofia Y Literatura*	Type
89	Taverniers, M.	2003	Grammatical metaphor in SFL: A historiography of the introduction and initial study of the concept	*Grammatical Metaphor: Systemic and Functional Perspectives*	Theoretical interpretation
90	Taverniers, M	2018	Grammatical metaphor and grammaticalization: The case of metaphors of modality	*Functions of Language*	Interdiscipline
91	Thompson, G.	2014	*Introducing Functional Grammar(3rd Edn.)*	Routledge	Theoretical

					interpretation
92	Tian, Y. H.	2017	On the semantic function of objectivity of grammatical metaphor	*Modern Foreign Languages*	Interdiscipline
93	Walldén, R.	2019	Scaffolding or side-tracking? The role of knowledge about language in content instruction	*Linguistics and Education*	Interdiscipline
94	Wang, C. L.	2015	The graduation of grammatical metaphor: The cline of ideational metaphor and interpersonal metaphor	*Foreign Languages and Their Teaching*	Semantic and Characteristic
95	Wang, F. F.	2013	Grammatical metaphor theory: Possible deconstruction	*Foreign Language Learning Theory and Practice*	Semantic and Characteristic
96	Wang, G. L.	2014	Rank shift of the projection system	*Foreign Languages Research*	Semantic and Characteristic
97	Wang, J.	2012	An examination of the clausal and discourse-level meanings of nominalization in grammatical metaphor	*Academic Forum*	Semantic and Characteristic
98	Wang, J. J.	2003	The manifestation of nominalization in discourse types	*Foreign Language Research*	Semantic and Characteristic
99	Wang, Y. Y., and Hu, G. W.	2025	Positive covariation or trade-off? A cross-disciplinary investigation of shell nouns and their congruent expressions in research articles	*Scientometrics*	Type
100	Wang, Z. H., and Wang, D. Y.	2020	From process, epithet to thingness: Nominalisation in English and Chinese	*Journal of Foreign Languages*	Semantic and Characteristic
101	Wei, Y. D.	2006	Grammar Metaphors and Their Significance to Cognitive Science	*Studies in Philosophy of Science and Technology*	Interdiscipline
102	Wei, Z. J.	2003	A contrastive study of metaphors of mood in English and Chinese	*Journal of Foreign Languages*	Type
103	Wei, Z. J.	2008	*A Comparative Study of Grammatical Metaphor in English and Chinese*	Foreign Language Teaching and Research Press	Type
104	Wei, Z. J.	2008	Metaphors of modality in English and Chinese: A contrastive study	*Modern Foreign Languages*	Semantic and Characteristic
105	Wu, L. C.	2024	On the relationship between semantic waves of ideational grammatical metaphor and knowledge construction in disciplinary discourse	*Foreign Language Teaching and Research*	Interdiscipline
106	Wu, L. C., and Zhang, D. L.	2023	A fractal study on ideational grammatical metaphor	*Modern Foreign Languages*	Interdiscipline
107	Yan, S. Q.	2003	The development of the grammatical metaphor theory and its theoretical implications	*Journal of Foreign Languages*	Theoretical interpretation
108	Yang, B.	2013	Ideational grammatical metaphor: A cognitive perspective	*Journal of Foreign Languages*	Interdiscipline
109	Yang, B.	2018	A probe into ideational grammatical metaphor	*Foreign Languages Research*	Theoretical interpretation
110	Yang, B. J.	2018	Interpersonal metaphor revisited: Identifification, categorization, and syndrome	*Social Semiotics*	Type

111	Yang, B. J.	2018	Textual metaphor revisited	*Australian Journal of Linguistics*	Type
112	Yang, B. J.	2019	Interpersonal metaphor revisited: identification, categorization, and syndrome	*Social Semiotics*	Type
113	Yang, B. J.	2020	*On Clause and Grammatical Metaphor*	Shanghai Foreign Language Education Press	Theoretical interpretation
114	Yang, B. J., and Gao, H. M.	2023	Polarity metaphor in English: Definition, identification, and categorization	*Lingua*	Type
115	Yang, C. Y.	2006	Metaphors of mood：A mechanism for expanding speech functions	*Foreign Languages Research*	Interdiscipline
116	Yang, X. Q.	2013	*Grammatical Metaphor and Semogenesis*	Nanjing University Press	Semantic and Characteristic
117	Yang, X. Q.	2013	*Halliday's Theory of Grammatical Metaphor and Semogenesis*	Suzhou University	Theoretical interpretation
118	Yang, Y. N.	2008	Typological interpretation of differences between Chinese and English in grammatical metaphor	*Language Sciences*	Semantic and Characteristic
119	Yang, Y. N.	2011	Grammatical metaphor in Chinese: A corpus-based study	*Functions of Language*	Type
120	Yang, Y. N.	2013	A corpus-based study of interpersonal grammatical metaphor in spoken Chinese	*Language Sciences*	Type
121	Yang, Y. N.	2020	*Research on Grammatical Metaphor in Chinese*	Peking University Press	Semantic and Characteristic
122	Yang, Z.	2022	*Exploring Grammatical Metaphor: Insights From Systemic Functional Linguistics*	Shanghai Foreign Language Education Press	Theoretical interpretation
123	Zeng, L.	2003	A study of the syntactic and semantic features of "Projection" from the perspective of ideational metaphor in SFL	*Modern Foreign Languages (Quarterly)*	Semantic and Characteristic
124	Zhang, B.	2015	German grammatical metaphors within the framework of cognitive grammar: Taking the interpersonal grammatical metaphor as an example	*Journal of PLA University of Foreign Languages*	Interdiscipline
125	Zhang, D. L., and Dong, J.	2014	On the development models of grammatical metaphor	*Foreign Language Teaching and Research*	Theoretical interpretation
126	Zhang, D. L., and Lei, Q.	2013	Research on grammatical metaphor in China	*Foreign Language Education*	others
127	Zhang, D. L., and Zhao, J.	2008	On the formal similarity principle in ideational grammatical metaphor between congruent and metaphorical forms	*Journal of Foreign Languages*	Semantic and Characteristic
128	Zhang, D. L., and Zhao, J.	2023	The function of grammatical metaphor in the construction of the semantic structure of discourse	*Foreign Language Education*	Theoretical interpretation
129	Zhang, F. K., and Shao, X. G.	2009	Cognitive motivations and discourse functions of English grammatical metaphor	*Shandong Foreign Language Teaching*	Interdiscipline
130	Zhao, D. Q., and Li, W.	2010	Metafunctions and grammatical metaphors	*Journal of Hebei University (Philosophy and Social Science)*	Theoretical interpretation

131	Zhao, L.	2016	*A Functional-Cognitive Study on Ideational Grammatical Metaphor at the Rank Scale of Clause*	Science Press	Interdiscipline
132	Zhao, L.	2018	The image schematic basis of metaphorical relational clauses in Halliday's sense	*Foreign Language Teaching and Research*	Interdiscipline
133	Zhong, S. N., and Fu, S. Y.	2016	Can quantitative adjectives in English really not be nominalized?	*Foreign Language Education*	Semantic and Characteristic
134	Zhou, J. P.	2023	A corpus-based study of explicit objective modal expressions in English	*Studla Neophilologica*	Semantic and Characteristic
135	Zhou, J. P.	2023	Complexity of grammatical metaphor: an entropy-based approach	*Semiotica*	Semantic and Characteristic
136	Zhou, J. P.	2024	Preferences of interpersonal metaphor of modality in academic disciplines	*Humanities & Social Sciences Communications*	Semantic and Characteristic
137	Zhou, J. P.	2025	Complexity of nominal group adjectivization: a self-information approach	*Semiotica*	Semantic and Characteristic
138	Zhou, N. N.	2025	Continua and orientations of packing-repacking and unpacking ideational metaphor for knowledge construction	*Poznan Studies in Contemporary Linguistics*	Interdiscipline
139	Zhou, P.	2008	A refutation of the claims of grammatical metaphor theory about science and relativism of truth	*Journal of Foreign Languages*	Interdiscipline
140	Zhu, Y. S.	2006	On nominalization, verbalization and grammatical metaphor	*Foreign Language Teaching and Research*	Theoretical interpretation
141	Zhu, Y. S., and Yan, S. Q.	2000	Contributions and limitations of Halliday's grammatical metaphor theory	*Foreign Language Teaching and Research （bimonthly）*	Theoretical interpretation

Table B: The studies within practical application

No.	Author(s)	Year	Title	Source	Sub-theme
1	Banks, D.	2003	The evolution of grammatical metaphor in scientific writing	*Grammatical Metaphor: Views from Systemic Functional Linguistics*	textual analysis
2	Bisiada, M.	2018	The editor's invisibility analysing editorial intervention in translation	*Target-International Journal of Translation Studies*	translation studies
3	Blunden, J.	2017	The sweet spot? Writing for a reading age of 12	*Curator-The Museum Journal*	language teaching
4	Bonnin, J. E.	2009	Religious and political discourse in Argentina: the case of reconciliation	*Discourse & Society*	textual analysis
5	Brdar, M., and Brdar-Szabó, R.	2017	On constructional blocking of metonymies: A cross-linguistic view	*Review of Cognitive Linguistics*	language teaching
6	Brookes, D. T., and Etkina, E.	2007	Using conceptual metaphor and functional grammar to explore how language used in physics	*Physical Review Special Topics-Physics Education Research*	language teaching

			affects student learning		
7	Castro, C., and Oteiza, T.	2022	Historical explanations in the Rettig Report: The role of interpersonal grammatical metaphors	*Discourse & Society*	textual analysis
8	Chai, G. Y., and Liu, J. L.	2019	Public image of local government: A transitive grammatical metaphor perspective	*Technology Enhanced Foreign Language Education*	textual analysis
9	Chen, J. W., and Gao, Y.	2014	Ideational grammatical metaphor and stylistic awareness in writing	*Journal of Xi'an International Studies University*	language teaching
10	Chen, M.	2013	A study on the functions of grammatical metaphor in scientific and technological discourse	*Journal of Hubei Minzu University (Philosophy and Social Sciences)*	textual analysis
11	Chen, Q.	2012	Scientific language: Metaphorical constructions and translation strategies	*Foreign Language Education*	translation studies
12	Chen, S. K., Mo, A. P., and Yang, S.	2024	"We should have a deep understanding": Reinstantiating cognitive processes in the translation of Chinese political discourse	*Perspectives — Studies in Translation Theory and Practice*	translation studies
13	Chen, Y. M., and Huang, G. W.	2014	On the readability of original literary work and simplified versions: A grammatical metaphor perspective	*Foreign Language Teaching and Research*	textual analysis
14	Christie, F., and Derewianka, B.	2008	*School D: Learning to Write Across the Years of Schooling*	Continuum	language teaching
15	Colombia, M. C.	2006	Grammatical metaphor: Academic language development in Latino students in Spanish	*Advanced Language Learning the Contribution of Halliday and Vygotsky*	language teaching
16	Deng, Y. R.	2013	Grammatical metaphor in English-Chinese translation	*Journal of Northeast Normal University (Philosophy and Social Sciences Edition)*	translation studies
17	Deng, Y. R., and Cao, Z. X.	2010	Translation as production: Congruent and metaphorical forms	*Foreign Language Research*	translation studies
18	Derewianka, B.	2003	Grammatical metaphor in the transition to adolescence	*Grammatical Metaphor: Views From Systemic Functional Linguistics*	language teaching
19	Dong, M.	2018	The cross-language logical grammar metaphorical perspective of interlingual realization	*Journal of PLA University of Foreign Languages*	translation studies
20	Dong, X. M., Lin, Z. J., and Zhang, H.	2024	Deployment of logical grammatical metaphor in academic discourse by Chinese EFL learners	*Foreign Language Education*	language teaching
21	Espunya, A.	2020	Reduced abstractness in Spanish-English translation: the case of property-denoting nouns	*Meta*	translation studies
22	Fan, R., and Hill-Madsen, A.	2025	Translation of medical concepts in West-East knowledge transmission in nineteenth century China: a case study of grammatical de-metaphorization	*Perspectives — Studies in Translation Theory and Practice*	translation studies

23	Fang, Y. G.	2021	*The Theory of Grammatical Metaphor and Its Application in English Teaching*	Atomic Energy Press	language teaching
24	Fusari, S.	2018	"Bacon wrapped cancer": The discursive construction of meat carcinogenicity	*Text & Talk*	textual analysis
25	Gamal, A.	2012	Postcolonial translation as transformation Ahdaf Soueif's I Think of You	*Translator*	translation studies
26	Galve, I.	1996	Grammatical metaphor across a medical journal: An initial analysis of the role of genre	*Current Issues in Genre Theory*	textual analysis
27	Galve, I.	1997	Is intertextuality behind the characteristic nominalizations of the medical journal article? Using grammatical metaphor as an intertextual signal	*The Intertextual Dimension of Discourse*	textual analysis
28	Galve, I.	2005	The use of ideational grammatical metaphor for interpersonal purposes in the medical research article	*Perspectivas Interdisciplinares de la Lingüística Aplicada*	textual analysis
29	Gao, C.	2015	An exploration of grammatical metaphor in the English news reports of "APEC Blue"	*Shandong Social Sciences*	textual analysis
30	Gao, H. M., Lu, C. M., and Hu, C. Y.	2024	A corpus-based study of signalling nouns in marketing and economics research articles	*Journal of English for Academic Purposes*	textual analysis
31	Gao, S. B.	2006	The metaphorical nature of texts and its implications for translation studies	*Foreign Languages and Their Teaching*	translation studies
32	Gao, W. Y.	2008	Application of nominalization in discussion sections of English medical research papers	*Foreign Languages and Their Teaching*	textual analysis
33	Glasson, N., and Kitney, A.	2025	Making things happen: A study of grammatical metaphors in L2 writing scripts	*Assessing Writing*	language teaching
34	Guijarro, A. J. M.	2016	The role of semiotic metaphor in the verbal-visual interplay of three children's picture books. A multisemiotic systemic-functional approach	*Atlantis*	textual analysis
35	Guinda, C. S., and Pellón, I. A.	2011	How patent can patents be? Exploring the impact of figurative language on the engineering patents genre	*Review of Cognitive Linguistics*	textual analysis
36	Guo, J. H.	2010	On nominalization in EST: Functions & cognitive effects	*Foreign Languages and Literature*	textual analysis
37	Guo, J. H., and L, W.	2025	The deployment and features of Chinese English Learners' logical grammatical metaphor: A corpus-based study of M. A. theses	*Journal of Foreign Languages*	language teaching
38	Hao, J.	2025	Nominalised activities in Chinese history texts: A systemic functional linguistic perspective	*Lingua*	textual analysis
39	Hao, J., and Humphrey, S. L.	2019	Reading nominalizations in senior science	*Journal of English for Academic Purposes*	textual analysis

40	He, Z. Q.	2023	Modal metaphors in mathematical English: Types, representations and distribution	*Journal of Xi'an International Studies University*	textual analysis
41	Heim, S.	2022	"I for i and i for I": Susan Howe's That This and the relational self	*Journal of Modern Literature*	others
42	Hu, G. W., and Perez, M. R.	2022	Effects of explicit instruction in nominalisation on ESL learners' academic writing	*Iberica*	language teaching
43	Huang, G. W.	2009	The analysis of grammatical metaphor in translation studies	*Chinese Translators Journal*	translation studies
44	Huang, G. W.	2018	Analyses of metafunctions and grammatical metaphors in nature-oriented poems: A case study of Emily Dickinson's "Nature" is what we see	*Foreign Language Education*	textual analysis
45	Jiang, S. J.	2008	Grammatical Metaphor in Technical English	*Social Sciences in Guangxi*	textual analysis
46	Juznic, T. M.	2013	Bridging a grammar gap with explicitation: A case study of the nominalized infinitive	*Across Languages and Cultures*	translation studies
47	Karlsson, A. M.	2009	Fixing meaning: On the semiotic and interactional role of written texts in a risk analysis meeting	*Text & Talk*	textual analysis
48	Klein, P. D., and Unsworth, L.	2014	The logogenesis of writing to learn: A systemic functional perspective	*Linguistics and Education*	language teaching
49	Koller, V., and Davidson, P.	2008	Social exclusion as conceptual and grammatical metaphor: A cross-genre study of British policy-making	*Discourse & Society*	textual analysis
50	Lai, L. T., and Su, S. M.	2025	The interpersonal rhetoric effect of interpersonal metaphor — A case study on literary interview discourse	*Contemporary Rhetoric*	textual analysis
51	Lee, J. S. Y., Cheung, L. M. E., Saberi, D., and Webster, J. J.	2019	Expanding students' registerial repertoire with a writing assistance tool	*Journal of English for Academic Purposes*	language teaching
52	Lejeune, P.	2018	The epistemic status of predictions in central bank reports: A cross-linguistic study	*International Journal of Business Communication*	textual analysis
53	Li, B. R., and Yang, B. J.	2024	A corpus-based study of nominalizations in the sports news of Shanghai Gazette (1919-1920)	*Word*	textual analysis
54	Li, H. B.	2010	An analysis of interpersonal meaning in advertising discourse from the perspective of mood metaphor	*Journal of Southwest University(Social Sciences Edition)*	textual analysis
55	Li, J.	2016	Grammatical metaphor theory in pursuit of metaphorical competence	*Foreign Languages in China*	language teaching
56	Li, J. X.	2009	The influence of grammatical metaphor input and its inadequacy on the awareness of register in Chinese EFL learners	*Journal of Sichun International Studies university*	language teaching

57	Li, W., and Guo, J. H.	2020	The ideational grammatical metaphor competence of Chinese advanced English learners: A corpus-based study of PhD theses	*Foreign Language Learning Theory and Practice*	language teaching
58	Liardét, C.L.	2013	An exploration of Chinese EFL learner's deployment of grammatical metaphor: Learning to make academically valued meanings	*Journal of Second Language Writing*	language teaching
59	Liardét, C. L.	2016	Nominalization and grammatical metaphor: Elaborating the theory	*English for Specific Purposes*	language teaching
60	Liardét, C. L.	2018	"As we all know": Examining Chinese EFL learners' use of interpersonal grammatical metaphor in academic writing	*English for Specific Purposes*	language teaching
61	Liardét, C. L.,and Black, S.	2020	Trump vs. Trudeau: Exploring the power of grammatical metaphor for academic communication	*Journal of English for Academic Purposes*	language teaching
62	Liardét, C. L., and Black, S.	2025	Decoding disciplinary expectations: an analysis of lecturers' benchmarks for success in undergraduate assignments	*Teaching in Higher Education*	language teaching
63	Liardét, C. L., Black, S., and Bardetta, V. S.	2019	Defining formality: Adapting to the abstract demands of academic discourse	*Journal of English for Academic Purposes*	textual analysis
64	Lin, F.	2002	Grammatical metaphor and its types comparison in English and Chinese scientific languages	*Journal of Xinjiang University (Philosophy and Social Sciences)*	textual analysis
65	Lim, E., and Kellogg, D.	2008	The ascent of the concrete: Grammatical reification in science teaching exchanges and episodes	*Language and Education*	language teaching
66	Liu, C. Y., and Tang, H. B.	2021	Literal language vs. legal language: Deconstructing the nominalized language in courtroom discourse	*Contemporary Rhetoric*	textual analysis
67	Liu, X.	2021	The low co-occurrence of nominalization and hedging in scientific papers written by Chinese EFL learners	*Arab World English Journal*	language teaching
68	Liu, Z. Y.	2008	Linkage of congruent form with metaphoric form in Chinese-to-English translation	*Journal of Tianjin University (Social Sciences)*	translation studies
69	Llinares, A., Morton, T., and Nashaat-Sobhy, N.	2025	An analysis of presence in CLIL students' writing when defining and exploring in science	*Journal of Research in Applied Linguistics*	language teaching
70	Lu, Y., and Pan, X. F.	2024	Writing performance and discourse organization in L2 Chinese: A longitudinal case study	*Journal of Second Language Writing*	language teaching
71	Magnusson, U.	2013	Grammatical metaphor in Swedish monolingual and multilingual upper secondary school students' writing	*Functions of Language*	language teaching

72	Marr, J. W.	2019	Making the mechanics of paraphrasing more explicit through Grammatical Metaphor	*Journal of English for Academic Purposes*	language teaching
73	Marr, J. W., and Mahmood, F.	2021	Looking past limiting conditions: Prioritizing meaning in EAP	*Journal of English for Academic Purposes*	language teaching
74	McGrath, D., and Liardét, C.	2022	A corpus-assisted analysis of grammatical metaphors in successful student writing	*Journal of English for Academic Purposes*	language teaching
75	McGrath, D., and Liardét, C.	2023	Grammatical metaphor across disciplines: Variation, frequency, and dispersion	*English for Specific Purposes*	textual analysis
76	McGrath, D., and Liardét, C.	2025	Grammatical metaphor across disciplines: Variation, frequency, and dispersion	*English for Specific Purposes*	language teaching
77	Miao, N.	2025	Semantic code construal and construction of disciplinary knowledge in educational discourse: A logical grammatical metaphor approach	*Foreign Languages in China*	textual analysis
78	Moss, G	2010	Textbook language, ideology and citizenship: The case of a history textbook in Colombia	*Functions of Language*	textual analysis
79	Mvanyashe, A.	2025	Nominalisation and semantic shift in selected isiXhosa nouns	*South African Journal of African Languages*	textual analysis
80	O'Toole, J. M., and King, R. A. R.	2010	A matter of significance: Can sampling error invalidate cloze estimates of text readability?	*Language Assessment Quarterly*	language teaching
81	Painter, C.	2003	The use of a metaphorical mode of meaning in early language development	*Grammatical Metaphor: Views From Systemic Functional Linguistics*	language teaching
82	Panza, C. B., Vuletich, L. J. G., Picchio, R. B., and Dichiara, A. K.	2024	Academic literacy in English: A pedagogical proposal for the teaching of nominalization in an undergraduate program	*Argentinian Journal of Applied Linguistics*	language teaching
83	Park, H.	2019	Grammatical metaphor in academic writing: Focusing on nominalization and verbalization	*The Journal of Linguistics Science*	language teaching
84	Petersen, D., and Almor, A.	2025	Agentive linguistic framing affects responsibility assignments toward AIs and their creators	*Frontiers in Psychology*	textual analysis
85	Pineh, A. J.	2022	Exploring nominalization use in EFL students' argumentative writing over a genre-based teaching and learning approach	*Journal of Research in Applied Linguistics*	language teaching
86	Rose, D.	2021	Reading metaphor: Symbolising, connoting and abducing meanings	*Linguistics and Education*	language teaching
87	Ryshina-Pankova, M.	2010	Toward mastering the discourses of reasoning: Use of grammatical metaphor at advanced levels of foreign language acquisition	*The Modern Language Journal*	language teaching
88	Ryshina-Pankova, M.	2011	Preparing graduate student teachers for advanced content-based instruction: Exploring content	*Educating the Future Foreign Language Professoriate for the*	language teaching

			through grammatical metaphor	*21st Century*	
89	Ryshina-Pankova, M.	2015	A meaning-based approach to the study of complexity in L2 writing: The case of grammatical metaphor	*Journal of Second Language Writing*	language teaching
90	Ryshina-Pankova, M., and Byrnes, H.	2013	Writing as learning to know: Tracing knowledge construction in L2 German Compositions	*Journal of Second Language Writing*	language teaching
91	Salum, N. L.	2022	Causal connexions in Spanish: a discourse semantics resource for explaining the recent past in school History	*Estudios Filologicos*	textual analysis
92	Shao, X. G., and Xu, X. Q.	2015	Cognitive effects of grammatical metaphor on English reading in multi-media setting	*Technology Enhanced Foreign Language Education*	language teaching
93	Sheldon, E.	2022	Construing FL writers' meaning-making choices in a historical recount genre in Spanish: A functional approach to developing academic literacy	*Language Context and Text — The Social Semiotics Forum*	language teaching
94	Shen, J. R.	2010	The working mechanism and functions of grammatical metaphor in news discourse	*Contemporary Rhetoric*	textual analysis
95	Song, L. Q., and Yang, Z.	2018	A study on the stylistic function of grammatical metaphor in contract English	*Foreign Languages in China*	textual analysis
96	Song, L. Q., and Yang, Z.	2019	On the motivation and stylistic function of interpersonal metaphor — A case study of interpersonal metaphor in business English letters	*Foreign Language Research*	textual analysis
97	Stenvall, M.	2008	On emotions and the journalistic ideals of factuality and objectivity — Tools for analysis	*Journal of Pragmatics*	textual analysis
98	Sun, C. R., and Song, D. S.	2008	An empirical study on the relationship between ideational grammatical metaphor and students' levels of text construction	*Foreign Language Research*	language teaching
99	Sun, Y. M., and Shao, X. G.	2011	Grammatical metaphor and English reading	*Journal of Southwest Minzu University (Humanities and Social Sciences Edition)*	language teaching
100	Tan, L. H.	2011	A corpus-based comparative study of the use of nominalization metaphor in scientific discourse	*Journal of Hunan University of Science and Technology(Social Science Edition)*	language teaching
101	Tang, B.	2007	Study on nominalization in advertising discourse	*Foreign Language Learning Theory and Practice*	textual analysis
102	Tang, H. B., and Liu, C. Y.	2021	Semantic fuzziness of nominalization in courtroom discourse and its elimination：A sociosemiotic perspective	*Modern Foreign Languages*	textual analysis
103	Thwaite, A., Budgen, F., Hunter, J., and	2021	What is complexity? Grammatical issues in assignment prompts	*Journal of English for Academic Purposes*	language teaching

	Singh, K.				
104	To, V.	2018	Linguistic complexity analysis: A case study of commonly-used textbooks in Vietnam	*Sage Open*	language teaching
105	To, V., Thomas, D., and Thomas, A.	2020	Writing persuasive texts: Using grammatical metaphors for rhetorical purposes in an educational context	*Australian Journal of Linguistics*	language teaching
106	Torr, J., and Simpson, A.	2003	*The Emergence of Grammatical Metaphor: Literacy Oriented Expressions in the Everyday Speech of Young Children*	John Benjamins	language teaching
107	Valdebenito, M. S.	2013	Image repair discourse of Chilean companies facing a scandal	*Discourse & Communication*	textual analysis
108	Walldén, R., and Larsson, P. N.	2021	Negotiating figurative language from literary texts: Second-language instruction as a dual literacy practice	*L1 Educational Studies in Language and Literature*	language teaching
109	Wang, B. J., Wang, B. P., and Wang, Y.	2020	The contribution of grammatical metaphor to the resources of second language writing research	*Foreign Language Learning Theory and Practice*	language teaching
110	Wang, D., Zhang, L., and Huang, Y.	2024	Tracking the development of logical metaphor usage in argumentative writing: A longitudinal study with EFL learners	*System*	language teaching
111	Wang, L. F., and Chen, G.	2008	A study of nominalization in English writing by college students	*Foreign Languages in China*	language teaching
112	Wang, R. Q.	2009	Grammatical metaphor and the entry of self-designation senses into Chinese dictionaries: A corpus-based study	*Foreign Languages and Literature*	others
113	Wang, Y., and Chen, Z. F.	2011	Metaphors of mood and its function in political discourse	*Journal of Anhui University (Philosophy and Social Sciences Edition)*	textual analysis
114	Wang, Z. H., and Shi, C. X.	2016	Nominalization and its effects on discourse	*Modern Foreign Languages*	textual analysis
115	Xiong, X. L., and Liu, D. H.	2005	Grammatical metaphors in English language learning	*Foreign Language Teaching and Research*	language teaching
116	Xu, X. Y.	2011	An analysis of the discourse functions of English nominalization: The case of international trade sales contracts	*Modern Foreign Languages*	textual analysis
117	Yahya, Y. K., Afandi, Z., and Burdah, I.	2023	Semantic adjustment in Matthew 6:12 in the Smith-Van Dyck Arabic Bible	*HTS Teologiese Studies — Theological Studies*	translation studies
118	Yang, B. J.	2019	Taishang zuozhe zhuxituan in Chinese as Ideational Grammatical Metaphor	*Foreign Languages in China*	others

119	Yang, J.	2013	A Comparison between English and Chinese textual metaphors in political speeches	*Journal of Nantong University (Social Sciences Edition)*	textual analysis
120	Yang, L.	2013	Nominalization in scientific English: Its functions in textual cohesion and its translation	*Chinese Science & Technology Translators Journal*	translation studies
121	Yang, W. T., Martin, R. M., and Wang, X. L.	2025	Revisiting grammatical metaphor in translation: New insights from the English-Chinese pair	*Metaphor and Symbol*	translation studies
122	Yang, X. Z.	2006	The role of nominalization in register: A small-scale corpus-based analysis	*Technology Enhanced Foreign Language Education*	others
123	Yasuda, S.	2015	Exploring changes in FL writers' meaning-making choices in summary writing: A systemic functional approach	*Journal of Second Language Writing*	language teaching
124	Ye, L., and Zeng, L.	2024	Grammatical demetaphorization in the title translation of Chinese political cartoons: A multimodal discourse analysis	*Translator*	translation studies
125	Yu, H., and Miao, N.	2020	Analysis of the patterns and types of causal logical grammatical metaphor in educational discourse	*Foreign Languages in China*	textual analysis
126	Yu, X. B.	2006	Nominalization and its inspiration on textual translation	*Journal of Guangxi Normal University for Nationalities*	translation studies
127	Zeng, L.	2007	"Projection" in academic discourse: From the perspective of grammatical metaphor	*Foreign Language Research*	textual analysis
128	Zeng, X. G.	2008	Reinterpreting grammatical metaphor in newspaper English	*Foreign Language Learning Theory and Practice*	textual analysis
129	Zhang, C. L.	2014	Analysis of grammatical metaphor in textual cohesion	*Academic Exchange*	textual analysis
130	Zhang, H., Lin, Z. J., and Dong, X. M.	2021	A study of English translation strategies of logical grammatical metaphor on report on the work of the government from the perspective of embodiment	*Foreign Language Research*	translation studies
131	Zhang, H., and Yang, B. J.	2025	"You want to know more about me?" A corpus-based study of symbolic violence realized by mood metaphors in videogame discourse	*Word-Journal of the International Linguistic Association*	textual analysis
132	Zhang, H. P., and Liu,Y. B.	2013	On English metaphorical preposition learning and ideational transfer under linguistic relativity principle	*Foreign Language Education*	language teaching
133	Zhao, D. Q., and Ning, Z. M.	2005	Interpretation of grammatical metaphor in English journalism	*Foreign Language Learning Theory and Practice*	textual analysis
134	Zhao, L.	2017	A BNC-based study on ideational grammatical metaphor at the clause rank	*Journal of PLA University of Foreign Languages*	others
135	Zhong, L. F., and Chen, X.	2015	An investigation into metaphor production in EAP writings	*Modern Foreign Languages*	language teaching

	H.				
136	Zhou, H., and Liu, Y. B.	2017	Investigating the use and discourse functions of grammatical metaphor in Chinese EFL learners' thesis abstracts	*Modern Foreign Languages*	language teaching
137	Zhu, C. S., and Zhang, J. F.	2015	Dancing with ideology: Grammatical metaphor and Identity presentation in translation	*Meta: Journal des Traducteurs/ Meta: Translators' Journal*	translation studies

Table C: Halliday's foundational works

No.	Author(s)	Year	Title	Source
1	Halliday, M. A. K.	1976	Anti-languages	*Language and Society*
2	Halliday, M. A. K.	1985	*An Introduction to Functional Grammar*	Arnold
3	Halliday, M. A. K.	1985	Dimension of discourse analysis: grammar	*On Grammar*
4	Halliday, M. A. K.	1988	On the language of physical science	*The Language of Science*
5	Halliday, M. A. K.	1989	Some grammatical problems in scientific English	*The Language of Science*
6	Halliday, M. A. K.	1993	Writing science: Literacy and discursive power	*The Language of Science*
7	Halliday, M. A. K.	1994	*An Introduction to Functional Grammar (2nd Ed.)*	Arnold
8	Halliday, M. A. K.	1995	Language and the reshaping of human experience	*The Language of Science*
9	Halliday, M. A. K.	1997	On the grammar of scientific English	*The Language of Science*
10	Halliday, M. A. K.	1998	Language and knowledge: The "unpacking" of text	*The Language of Science*
11	Halliday, M. A. K.	1998	Things and relations: re-grammaticizing experience as technical knowledge	*The Language of Science*
12	Halliday, M. A. K.	1999	The grammatical construction of scientific knowledge: The framing of the English clause	*The Language of Science*
13	Halliday, M. A. K., and C.M. I. M. Matthiessen	1999	*Construing Experience Through Meaning: A Language-Based Approach to Cognition*	Continuum
14	Halliday, M. A. K., and C. M. I. M. Matthiessen	2004	*An Introduction to Functional Grammar (3rd Ed.)*	Arnold
15	Halliday, M. A. K., and C. M. I. M. Matthiessen	2014	*An Introduction to Functional Grammar (4rd Ed.)*	Routledge
